# The Association Between the Baseline and the Change in Neutrophil-to-Lymphocyte Ratio and Short-Term Mortality in Patients With Acute Respiratory Distress Syndrome

**DOI:** 10.3389/fmed.2021.636869

**Published:** 2021-05-14

**Authors:** Wei Zhang, Yadan Wang, Weijie Li, Guizuo Wang

**Affiliations:** ^1^Department of Respiratory and Critical Care Medicine, Shaanxi Provincial People's Hospital, Xi'an, China; ^2^Ruibiao (Wuhan) Biotechnology Co. Ltd., Wuhan, China

**Keywords:** change, neutrophil to lymphocyte ratio, NLR, ARDS, 30-day mortality, intensive care unit

## Abstract

**Background:** Two previous studies have shown that increased neutrophil to lymphocyte ratio (NLR) is associated with short-term prognosis in patients with acute respiratory distress syndrome (ARDS), but it is usually assessed as a single threshold value at baseline. We investigated the relationship between the baseline and the early change in NLR and 30-day mortality in patients with ARDS to evaluate the prognostic value of NLR baseline and NLR changes during the first 7 days after ICU admission.

**Methods:** This is a retrospective cohort study, with all ARDS patients diagnosed according to the Berlin definition from the Medical Information Mart for Intensive Care III (MIMIC-III) database. We calculated the NLR by dividing the neutrophil count by the lymphocyte count. The multivariable logistic regression analysis was used to investigate the relationship between the baseline NLR and short-term mortality. Then the generalized additive mixed model was used to compare trends in NLR over time among survivors and non-survivors after adjusting for potential confounders.

**Results:** A total of 1164 patients were enrolled in our study. Multivariable logistic regression analysis showed that after adjusting for confounders, elevated baseline NLR was a significant risk factor predicting 30-day mortality (OR 1.02, 95%CI 1.01, 1.03, *P* = 0.0046) and hospital mortality (OR 1.02, 95%CI 1.01, 1.03, *P* = 0.0003). The result of the generalized additive mixed model showed that the NLR decreased in the survival group and increased in the non-survival group gradually within 7 days after ICU admission. The difference between the two groups showed a trend of increase gradually and the difference increased by an average of 0.67 daily after adjusting for confounders.

**Conclusions:** We confirmed that there was a positive correlation between baseline NLR and short-term mortality, and we found significant differences in NLR changes over time between the non-survival group and the survival group. The early increase in NLR was associated with short-term mortality in ARDS patients.

## Introduction

Acute respiratory distress syndrome (ARDS) is a fatal form of acute respiratory failure requiring mechanical ventilation, which is caused by direct (pneumonia or aspiration) or indirect lung injury (sepsis or trauma) (1). It is a type of acute diffuse inflammatory lung injury that results in damage to the pulmonary endothelium, increased capillary permeability, pulmonary edema formation, and thus leads to decreased effective pulmonary ventilation area (2). Despite the efforts in early diagnosis and treatment, to our knowledge, there is no available treatment which can aim directly at the pathological mechanism of ARDS, and mechanical ventilation and supportive care are still the main approaches (1, 3). Approximately 35–46% of patients died consequently during hospitalization, making it one of the most common causes of death in intensive care units (ICU) (4). Therefore, it is urgent to find specific biological markers to accurately identify high-risk patients and adjust clinical treatment and nursing intervention to ultimately improve the prognosis of patients with ARDS.

Inflammatory response plays an important role in the development of ARDS, and is also an important factor affecting the prognosis of ARDS patients (2, 5). *In vivo* and *in vitro* studies have shown that inflammatory response can cause increased permeability of alveolar endothelium and epithelial cells, leading to the occurrence of pulmonary edema (5, 6). Neutrophil lymphocyte ratio (NLR) as a marker of systemic inflammation has been shown to be associated with the prognosis of a variety of diseases, including sepsis (7), COVID-19 pneumonia (8, 9), chronic obstructive pulmonary disease (COPD) (10), acute coronary syndrome (11), and several solid tumors (12–14). Two previous studies have shown that elevated baseline NLR was associated with short-term prognosis in ARDS patients (15, 16). However, these two studies only revealed the relationship between static NLR at baseline and short-term patient prognosis, which may not reflect the overall dynamics of the patient's condition. In this study, we investigated the relationship between baseline and early changes within the first week after ICU admission in NLR and short-term prognosis among ARDS patients.

## Patients and Methods

### Data Sources

This is a retrospective cohort study of 1164 patients with ARDS according to the Berlin definition. We obtained all the data from the Medical Information Mart for Intensive Care III (MIMIC-III), which is a single-center and freely accessible database and contains more than 60,000 health-related hospitalizations from 2001 to 2012 in the ICU of Beth Israel Deaconess Medical Center in Boston. We have completed the online course and passed the online exams (no. 6182750) to gain access to the database. The establishment of the MIMIC III database was approved by the institutional review board of Beth Israel deacons Medical Center and Massachusetts Institute of Technology. Because hospitalization information is anonymous, the informed consent was not required.

### Patients

We screened all the patients in the database. Our inclusion criteria were as follows: at least 16 years, ICU stay more than 72 h, ARDS diagnosis meeting Berlin standard at the time of ICU admission, had lymphocyte and neutrophil count within 24 h after ICU admission. We excluded patients with chronic hematologic disorders, lacked the baseline of lymphocyte and neutrophil records. If a patient was admitted repeatedly during the study period, we used only the record of his first ICU admission.

The Berlin standard included: acute onset, arterial oxygen partial pressure (PaO_2_)/fraction of inspired oxygen (FiO_2_) <300 mmHg and positive end-expiratory pressure (PEEP) ≥5 cm H_2_O on the first day of ICU admission, bilateral infiltrates on chest radiograph, absence of heart failure. According to the Berlin standard, ARDS were classified into mild (>200 mmHg, ≤300 mmHg), moderate (>100 mmHg, ≤ 200 mmHg), and severe (<100 mmHg) based on the PaO_2_/FiO_2_ ratio.

### Data Extraction

We used the Structured Query Language to extract the data. We extracted or calculated the following variables, including the baseline characteristics (age, gender, ethnicity, admission type), the patients' comorbidity, the Elixhauser Comorbidity Index (SID30), the risk factors leading to ARDS, the vital signs within 24 h after ICU admission (heart rate, temperature, mean arterial pressure, Spo2), the severity of organ dysfunction (Simplified Acute Physiology Score, SAPS II; Oxford Acute Severity of Illness Score, OASIS; Sequential Organ Failure Assessment, SOFA), PaO_2_/FiO_2_ at diagnosis, PEEP at diagnosis and the treatment received (ventilation received; vasopressor therapy; renal replacement therapy; corticosteroids therapy; antibiotic therapy). The serious score of organ dysfunction was estimated for all patients within 24 h of ICU admission.

The baseline of NLR was determined by the absolute neutrophil count divided by the absolute lymphocyte count based on the first laboratory parameters after ICU admission. Repeated measurements of NLR were performed during the first 7 days after ICU admission. The repeated measurements of NLR were irregularly spaced over time.

### Statistical Analysis

We processed and analyzed all the data by EmpowerStats software (www.empowerstats.com version R.3.4.3) and statistical software package R. We presented the continuous variables as the mean (SD) and compared them by Student's *t*-test (normal distribution) or Mann-Whitney test (non-normal distribution); we presented the categorical variables as percentages and compared them by chi-square test. A two-tailed *P* < 0.05 was considered statistically significant.

We first analyzed the relationship between the baseline NLR and short-term clinical outcomes in ARDS patients. We divided the patients into three groups according to the tertile of the baseline NLR values. We used multivariable logistic regression analysis and smooth curve fitting to test the independent effects of the baseline NLR and in-hospital and 30-day mortality with crude and full models. The adjusted variables included age, gender, ethnicity, smoking, admission type, COPD, hypertension, tumor, diabetes mellitus, renal failure, SID30, pneumonia, sepsis, aspiration, trauma/surgery, other non-pulmonary, PaO_2_/FiO_2_, SAPS II, OASIS, SOFA, corticosteroids therapy, antibiotic therapy, vasopressor therapy, ventilation received and renal replacement therapy. We selected those confounders based on their associations with the outcomes of interest or a change in effect estimate of more than 10%.

Then we analyzed the relationship between the early change of NLR and 30-day mortality. We showed the difference in NLR between survivors and non-survivors within the first 7 days after ICU admission. We used the generalized additive mixed model (GAMM) to investigate the early changes of NLR over time between survivors and non-survivors, with crude and full models. The GAMM is commonly used to analyze the results of repeated measurements, especially when the interval between repeated measurements is irregular and some data is missing (17, 18).

## Results

### Characteristics of Patients

**Figure 1:**
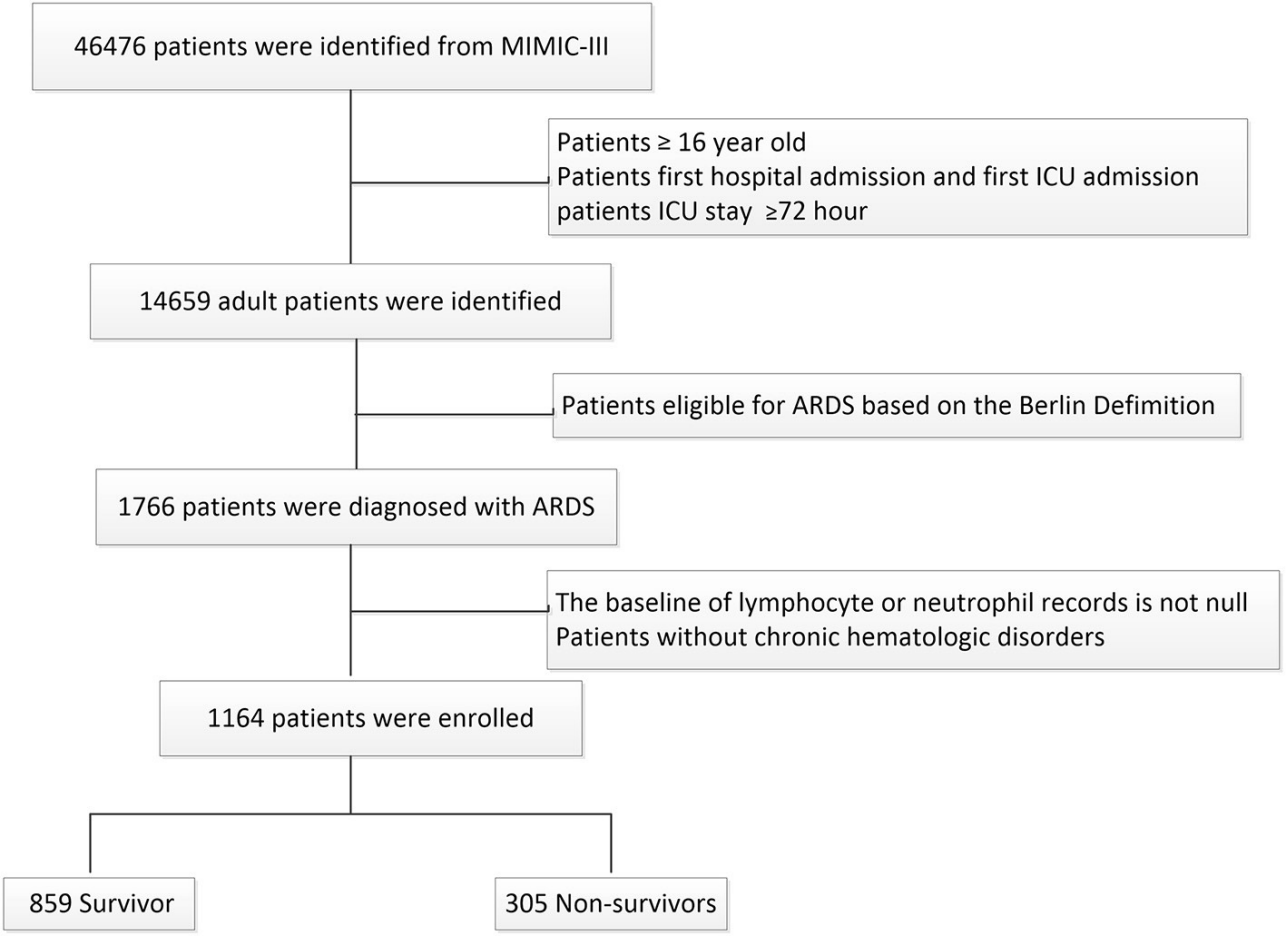
Flow chart of the current study.

A total of 1164 patients with ARDS were included in this study, which contained 859 survivors and 305 non-survivors who were stratified on 30-day mortality. The flowchart of study cohort selection was shown in Figure 1. The characteristics of the study participants were displayed in Table 1.

**Table 1:** Baseline characteristics of total cohort, 30-day survivors, and 30-day non-survivors.

**Variables**	**Total cohort**	**30-day survivor**	**30-day non-survivors**	***P*-value**
	***N* = 1164**	***N* = 859**	***N* = 305**	
Age (years)	60.27 ± 17.74	58.23 ± 17.53	66.03 ± 17.06	<0.001
Female	479 (40.87%)	346 (40.28%)	129 (42.30%)	0.538
Ethnicity				<0.001
Caucasian	782 (67.18%)	606 (70.55%)	176 (57.70%)	
Black	64 (5.50%)	48 (5.59%)	16 (5.25%)	
Hispanic	30 (2.58%)	26 (3.03%)	4 (1.31%)	
Others	288 (24.74%)	179 (20.84%)	109 (35.74%)	
Smoking, *n* (%)	595 (51.12%)	452 (52.62%)	143 (46.89%)	<0.001
Admission type				0.033
Emergency	1021 (87.71%)	744 (86.61%)	277 (90.82%)	
Urgent	94 (8.08%)	80 (9.31%)	14 (4.59%)	
Elective	49 (4.21%)	35 (4.07%)	14 (4.59%)	
**Risk factor**, ***n*** **(%)**				
Pneumonia	345 (29.64%)	235 (27.36%)	110 (36.07%)	0.004
Non-pulmonary sepsis	549 (47.12%)	390 (45.40%)	159 (52.13%)	0.043
Aspiration	77 (6.62%)	56 (6.52%)	21 (6.89%)	0.825
Trauma/surgery	293 (25.17%)	250 (29.10%)	43 (14.10%)	<0.001
Other non-pulmonary	328 (28.18%)	236 (27.47%)	92 (30.16%)	0.370
**Comorbidity**				
COPD	61 (5.24%)	43 (5.01%)	18 (5.90%)	0.546
Tumor	78 (6.70%)	39 (4.54%)	39 (12.79%)	0.001
Renal failure	140 (12.03%)	98 (11.41%)	42 (13.77%)	0.276
Hypertension	121 (10.40%)	92 (10.71%)	29 (9.51%)	0.555
Diabetes mellitus	272 (23.37%)	204 (23.75%)	68 (22.30%)	0.606
SID30	8.51 ± 7.58	7.73 ± 7.40	10.70 ± 7.63	<0.001
**Severity scale**				
SAPSII	44.36 ± 15.02	41.89 ± 13.79	51.30 ± 16.16	<0.001
OASIS	38.86 ± 8.08	37.92 ± 7.81	41.50 ± 8.25	<0.001
SOFA	7.20 ± 3.65	6.81 ± 3.35	8.30 ± 4.22	<0.001
**Baseline vital data**				
Temperature, °C	37.12 ± 0.79	37.20 ± 0.76	36.89 ± 0.81	<0.001
HR, beats/min	91.40 ± 17.28	91.66 ± 17.21	90.64 ± 17.49	0.375
MAP mmHg	76.52 ± 9.63	77.42 ± 9.58	73.98 ± 9.33	<0.001
Spo2	96.82 ± 2.95	97.01 ± 2.84	96.30 ± 3.18	<0.001
PaO_2_/FiO_2_ at diagnosis	136.57 ± 64.37	140.08 ± 63.98	126.69 ± 64.54	<0.001
PEEP at diagnosis	8.33 ± 13.57	7.91 ± 3.96	9.51 ± 25.65	0.076
NLR at diagnosis	14.10 ± 11.54	13.14 ± 10.66	16.78 ± 13.38	<0.001
Berlin classification, *n* (%)				0.007
Mild	208 (17.87%)	162 (18.86%)	46 (15.08%)	
Moderate	534 (45.88%)	408 (47.50%)	126 (41.31%)	
Severe	422 (36.25%)	289 (33.64%)	133 (43.61%)	
**Treatment received**				
Ventilation received	1032 (88.66%)	776 (90.34%)	256 (83.93%)	0.002
Vasopressor therapy	586 (50.34%)	426 (49.59%)	160 (52.46%)	0.390
Renal replacement therapy	74 (6.36%)	57 (6.64%)	17 (5.57%)	0.514
Corticosteroids therapy	87 (7.47%)	62 (7.22%)	25 (8.20%)	0.576
Antibiotic therapy	549 (47.16%)	444 (51.69%)	105 (34.43%)	<0.001

*Data were mean ± SD, n (%). p-values comparing groups were from Student's t-test or Mann-Whitney test for continuous data and chi-squared test for categorical variables*.

*COPD, chronic obstructive pulmonary disease; SID30, elixhauser comorbidity score; MAP, mean arterial pressure; HR, heart rate; PaO_2_/FiO_2_, arterial oxygen partial pressure/ fraction of inspired oxygen; SAPSII, Simplified Acute Physiology Score; OASIS, Oxford Acute Severity of Illness Score; SOFA, Sequential Organ Failure Assessment; PEEP, positive end-expiratory pressure*.

The average age of the study participants was 60.27 ± 17.74 years and 40.87% of participants were female. The main risk factors of ARDS were non-pulmonary sepsis (*N* = 549 47.12%) and pneumonia (*N* = 345 29.64%). There were 208 (17.87%), 534 (45.88%), and 422 (36.25%) patients with mild, moderate and severe ARDS, respectively, upon ICU admission. The median values of NLR at baseline were 13.14 ± 10.66 in survivors and 16.78 ± 13.38 in non-survivors, respectively. We found that there was a significant difference between the two groups in terms of age, ethnicity, smoking and admission type; The SAPS II, OASIS, and SOFA scores within 24 h of ICU admission were significantly higher in non-survivors than that in survivors. There were also significant differences in vital signs (temperature, mean arterial pressure, Spo2, and PaO_2_/FiO_2_ at diagnosis) and treatment received (mechanical ventilation and antibiotic therapy).

### Association Between Baseline NLR and Mortality

We divided all the patients into three groups according to the tertiles of baseline NLR and Table 2 displayed the clinical outcomes of the subjects across the tertile of baseline NLR. Patients with high NLR (NLR ≥14.8) had shorter days free of mechanical ventilation, higher in-hospital, and 30-day mortality.

**Table 2:** Outcomes of the patients with ARDS across tertile of the baseline NLR.

**Variables**	**All patients**	**NLR**			***P*-value**
		**<7.5**	**≥7.5, <14.8**	**≥14.8**	
*n*	1,164	388	388	388	
Time in ICU (days)	15.53 ± 13.13	15.13 ± 12.20	15.81 ± 12.80	15.63 ± 14.33	0.757
Time in hospital (days)	22.34 ± 16.68	22.56 ± 17.67	22.25 ± 14.94	22.21 ± 17.34	0.950
Days free of MV at day 30	15.37 ± 11.07	16.39 ± 10.62	16.54 ± 10.69	13.19 ± 11.57	<0.001
In-hospital mortality	336 (28.87%)	91 (23.45%)	103 (26.55%)	142 (36.60%)	<0.001
30-day mortality	305 (26.20%)	82 (21.13%)	91 (23.45%)	132 (34.02%)	<0.001

*Data were mean ± SD, n (%). p-values comparing groups were from Student's t-test for continuous data and chi-squared test for categorical variables*.

**Figure 2:**
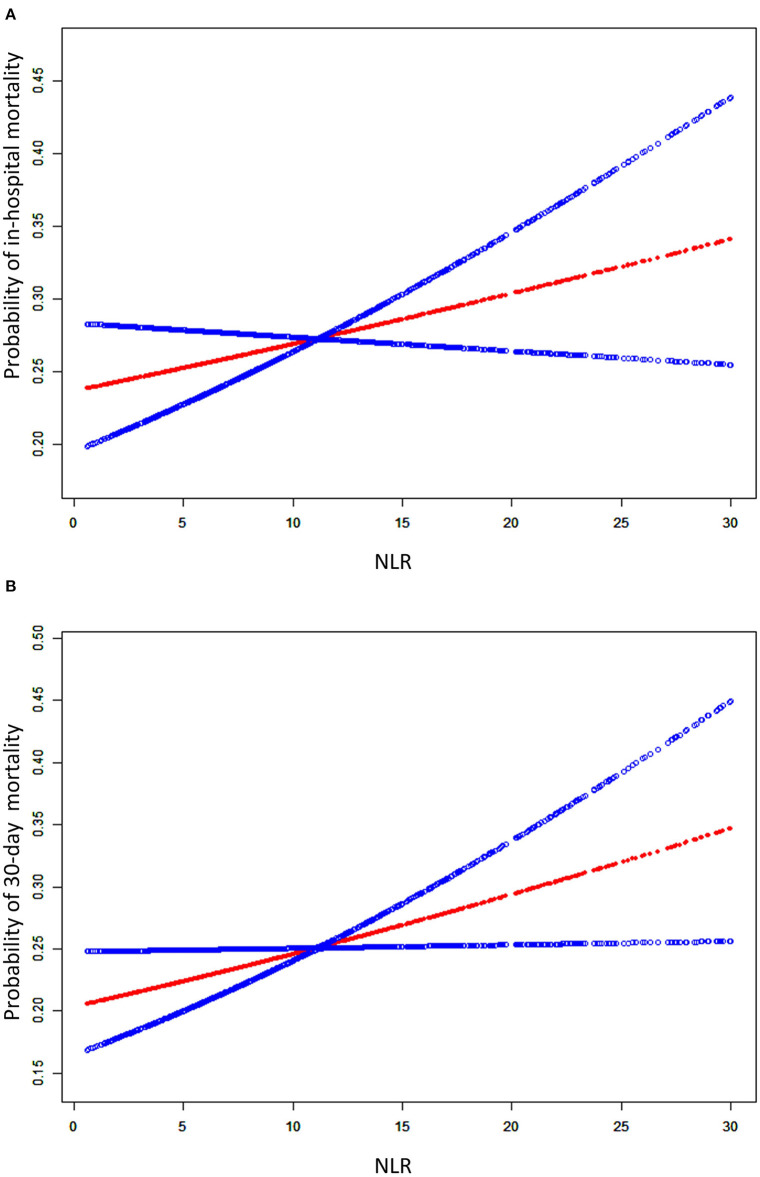
A smooth curve fitting for the relationship between the baseline NLR and the risk of in-hospital mortality **(A)** and 30-day mortality **(B)**. The resulting figures show the risk of mortality in the y-axis and the NLR (continuous variable) in the x-axis. The red line shows the dose-response curve between NLR and probability of short-term mortality, the two blue lines refer to 95% CIs. A positive relationship between NLR and the risk of short-term mortality was observed after adjusting for age, gender, ethnicity, smoking, admission type, COPD, hypertension, tumor, diabetes mellitus, renal failure, SID30, pneumonia, sepsis, aspiration, trauma/surgery, other non-pulmonary, PaO_2_/FiO_2_, SAPS II, OASIS, SOFA, corticosteroids therapy, antibiotic therapy, vasopressor therapy, ventilation received, and renal replacement therapy by spline smoothing plot.

We used the smooth curve fitting to show the association between baseline NLR levels and risk of in-hospital and 30-day mortality which was presented in Figure 2. Taking NLR on admission as a continuous variable, there was a significant positive association with the short-term mortality in the adjusted model.

We further evaluated this finding by the multivariable logistic regression analysis, which was shown in Table 3. As a continuous variable, after adjusting for the clinical confounders listed, an SD increase in baseline NLR levels was associated with a 2% higher risk of 30-day mortality (OR 1.02, 95% CI, 1.01, 1.03, *P* = 0.0046), and there was a similar trend for in-hospital mortality (OR 1.02, 95% CI, 1.01, 1.03, *P* = 0.0004). When RDW was assessed as tertiles, we found that patients in high baseline NLR (NLR ≥14.8) also had significantly higher risks of in-hospital (OR 1.48, 95% CI 1.03–2.12, *P* = 0.0319) and 30-day (OR 1.48, 95% CI 1.02–2.16, *P* = 0.0409) mortality than patients in low group (NLR <7.5) in the adjusted model.

**Table 3:** Multivariable logistic regression analysis of baseline NLR for mortality.

**Exposure**	**Non-adjusted**			**Adjust I**			**Adjust II**		
**30-day mortality**	**OR**	**95% CI**	***p*-value**	**OR**	**95% CI**	***p*-value**	**OR**	**95% CI**	***p*-value**
NLR Per 1 sd	1.03	(1.01, 1.04)	<0.0001	1.02	(1.01, 1.03)	0.0003	1.02	(1.01, 1.03)	0.0046
**NLR tertile**									
T1	1.0	(Reference)		1.0	(Reference)		1.0	(Reference)	
T2	1.14	(0.82, 1.60)	0.4378	1.07	(0.75, 1.53)	0.6921	1.09	(0.74, 1.60)	0.6643
T3	1.92	(1.39, 2.65)	<0.0001	1.74	(1.24, 2.45)	0.0015	1.48	(1.02, 2.16)	0.0409
**In-hospital mortality**	**OR**	**95% CI**	***p*****-value**	**OR**	**95% CI**	***p*****-value**	**OR**	**95% CI**	***p*****-value**
NLR Per 1 sd	1.03	(1.02, 1.04)	<0.0001	1.02	(1.01, 1.04)	<0.0001	1.02	(1.01, 1.03)	0.0004
**NLR tertile**									
T1	1.0	(Reference)		1.0	(Reference)		1.0	(Reference)	
T2	1.18	(0.85, 1.63)	0.3201	1.11	(0.78, 1.54)	0.5510	1.15	(0.80, 1.66)	0.4544
T3	1.88	(1.38, 2.58)	<0.0001	1.71	(1.23, 2.39)	0.0014	1.48	(1.03, 2.12)	0.0319

*OR, odds ratio; CI, confidence interval*.

*Adjusted I for age, gender, ethnicity, smoking, admission type*.

*Adjusted II for age, gender, ethnicity, smoking, admission type, COPD, hypertension, tumor, diabetes mellitus, renal failure, SID30, pneumonia, sepsis, aspiration, trauma/surgery, other non-pulmonary, PaO_2_/FiO_2_, SAPSII, OASIS, SOFA, corticosteroids therapy, antibiotic therapy, vasopressor therapy, ventilation received, and renal replacement therapy*.

The subgroup analyses for the relationship between baseline NLR and mortality were presented in Supplementary Table 1, which were performed according to age, gender, race, smoking, type of admission, risk factor, comorbidity, Berlin classification, and treatment received. The results showed that in different subgroups the relationship between baseline NLR and the risk of mortality stably existed except for patients of other race.

### Association Between Early Change in NLR and Mortality

We compared the difference in NLR between 30-day survivors and 30-day non-survivors on admission, on the 2–3th, the 4–5th, and the 6–7th day. We found that there were significant differences in NLR between the survival and the non-survival at the time points mentioned above. The result was shown in Table 4.

**Table 4:** The evolution of NLR after ICU admission between 30-day survivors and 30-day non survivors.

**Time**	**NLR, mean (SD) median (25th−75th percentile)**		
	**Survivors**	**Non-survivors**	***P*-value**
On admission	13.14 (10.66), 9.89 (5.97–16.40)	16.78 (13.38), 12.67 (6.96–22.32)	<0.001
On 2–3th day	11.97 (9.16), 9.08 (6.15–15.48)	17.39 (15.09), 13.33 (6.87–20.74)	<0.001
On 4-5th day	12.52 (9.31), 9.66 (5.86–15.70)	18.68 (19.04), 12.09 (8.06–20.53)	<0.001
On 6–7th day	11.73 (9.89), 9.25 (5.90–14.11)	21.25 (22.87), 13.56 (8.21–21.83)	<0.001

*p-value: as for the difference between survivors and non-survivors; Mann-Whitney test was applied for the variables with a skewed distribution*.

**Figure 3:**
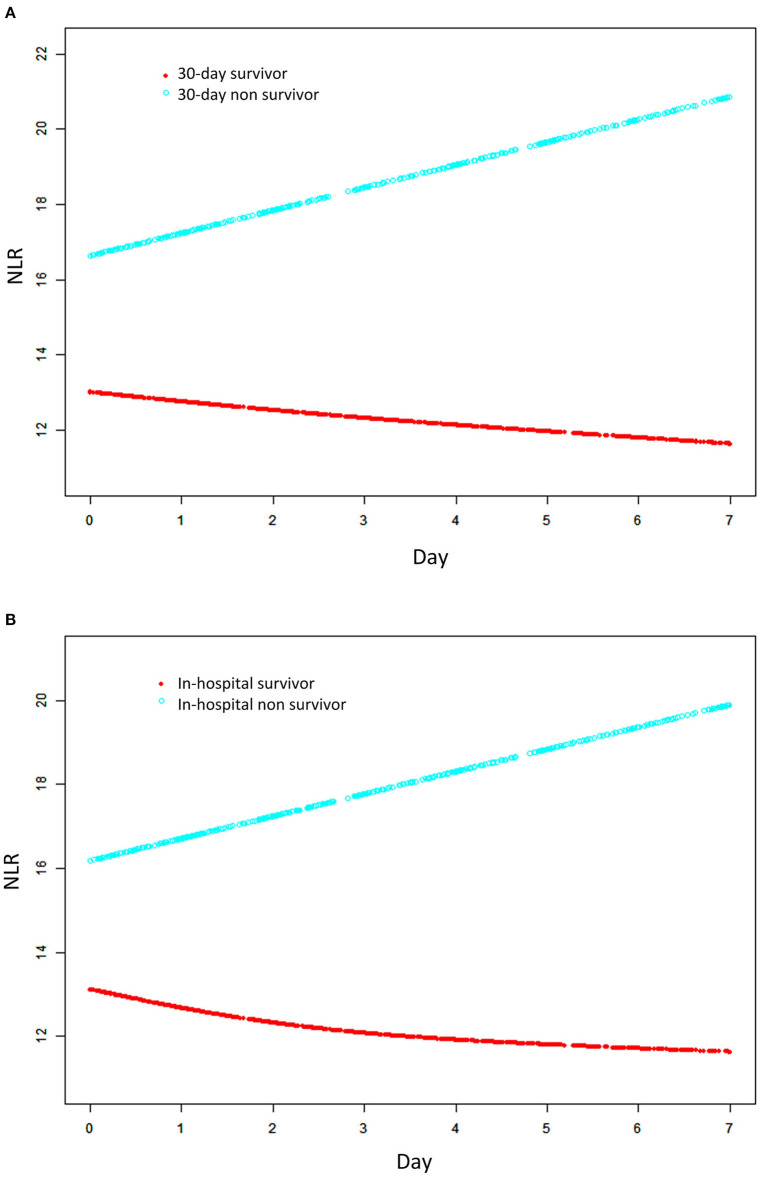
Association between changes in NRL and 30-day mortality **(A)** and in-hospital mortality **(B)**. A linear association between changes in NLR and mortality was found in a generalized additive mix model (GAMM). Smooth curve fitting graph illustrated the NLR in 1164 ARDS patients based on the days after admission to ICU. The red line represented the survivors. The blue line represented the non-survivors. All adjusted for age, gender, ethnicity, smoking, admission type, COPD, hypertension, tumor, diabetes mellitus, renal failure, SID30, pneumonia, sepsis, aspiration, trauma/surgery, other non-pulmonary, PaO_2_/FiO_2_, SAPS II, OASIS, SOFA, corticosteroids therapy, antibiotic therapy, vasopressor therapy, ventilation received, and renal replacement therapy.

We used GAMM to show the changes in NLR over time (the first week after ICU admission) between 30-day survivors and 30-day non-survivors after adjusted for clinical confounders listed. The result was presented in Figure 3. We found that, as time went on, NLR increased in the non-survival group. While the trend was reversed and it decreased in the survival group gradually within 1 week after ICU admission. And the difference between the two groups increased over time.

Furthermore, we revealed the association between early changes in NLR and 30-day mortality in ARDS patients which was presented in Table 5. The results indicated that NLR in the non-survival group was significantly higher than that in the survival group, and more importantly the difference between the two groups showed an increasing trend within 1 week after ICU admission. This difference increased by an average of 0.69 per day, and the results were consistent in the adjusted model (β = 0.67, 95% CI 0.23 1.11, *P* = 0.0030). These trends were also observed for in-hospital mortality.

**Table 5:** Relationship between changes (0–7 days) NLR and short-term mortality in patients with ARDS derived from a generalized additive mixed model (GAMM).

	**Unadjusted**		**Adjusted I**		**Adjusted II**	
**30-day mortality**	**β (95%CI)**	***P*-value**	**β (95%CI)**	***P*-value**	**β (95%CI)**	***P*-value**
Day	−0.23 (−0.46, −0.001)	0.0496	−0.23 (−0.46, 0.01)	0.0562	−0.25 (−0.48, −0.02)	0.0358
Death	3.86 (2.43, 5.28)	<0.0001	3.43 (1.97, 4.88)	<0.0001	2.64 (1.09, 4.19)	0.0009
Day × death	0.69 (0.25, 1.13)	0.0022	0.68 (0.24, 1.12)	0.0026	0.67 (0.23, 1.11)	0.0030
**In-hospital mortality**	**β** **(95%CI)**	***P*****-value**	**β** **(95%CI)**	***P*****-value**	**β** **(95%CI)**	***P*****-value**
Day	−0.30 (−0.54, −0.06)	0.0132	−0.29 (−0.53, −0.06)	0.0159	−0.31 (−0.55, −0.07)	0.0103
Death	3.38 (2.00, 4.77)	<0.0001	2.98 (1.56, 4.39)	<0.0001	2.22 (0.76, 3.73)	0.0032
Day × death	0.75 (0.33, 1.18)	0.0005	0.74 (0.32, 1.16)	0.0006	0.73 (0.31, 1.15)	0.0007

*CI, confidence interval; Day, the mean of the increasing of NLR at death = 0 over time (daily); Death, the difference of NLR at day = 0 between the group of death = 1 and the group of death = 0; Day × death, the average increasing in NLR daily under the condition of the group of death = 1 compared with the group of death = 0*.

*Adjusted I for age, gender, ethnicity, smoking, admission type*.

*Adjusted II for age, gender, ethnicity, smoking, admission type, COPD, hypertension, tumor, diabetes mellitus, renal failure, SID30, pneumonia, sepsis, aspiration, trauma/surgery, other non-pulmonary, PaO_2_/FiO_2_, SAPSII, OASIS, SOFA, corticosteroids therapy, antibiotic therapy, vasopressor therapy, ventilation received, and renal replacement therapy*.

## Discussion

In this retrospective cohort study, we confirmed that there was a significant positive correlation between baseline NLR and short-term mortality of patients with ARDS. High NLR at ICU admission was associated with higher 30-day and in-hospital mortality. Moreover, the NLR dynamic during the first 7-days after ICU admission also appears to be a good prognostic factor in patients with ARDS. We found that NLR increased gradually within 7 days after ICU admission in the non-survival group, but decreased gradually in the survival group. Furthermore, this difference between the two groups increased significantly over time. The relationship remained stable after adjusting for clinical confounders. This might suggest that an early increase in NLR is associated with poor short-term prognosis in ARDS patients.

Previous studies have shown that increased NLR was associated with short-term prognosis in a variety of diseases, including tumors, cardiovascular disease, and inflammatory diseases (7, 8, 10–12, 14, 19, 20). To our knowledge, there are only two previous studies on the relationship between baseline NLR and clinical outcomes of ARDS. The results of these two studies both showed that increased NLR on admission was associated with poor outcomes of ARDS patients. Wang et al. suggested that NLR >14 can be used as the cut-off threshold for predicting the 28-day mortality in patients with ARDS (16). Li et al. found that combining NLR could improve the prognostic value of APACHE II score and Berlin classification than using any of them alone (15). Our results for the relationship between baseline NLR and short-term prognosis in ARDS patients were consistent with previous studies. However, there is no study to explore the association between an early change of NLR and mortality in ARDS patients, which may reflect the comprehensive dynamic changes of the patient's condition and the evolution of the disease.

Therefore, we first used the GAMM model to explore the temporal variation of NLR in ARDS patients and its relationship with short-term outcomes. First, our study showed that NLR changed over time in ARDS patients within the first 7 days after ICU admission. Second, we compared the different trends of NLR over time between survival and death groups. Finally, the GAMM model was used to investigate the relationship between early changes in NLR and short-term prognosis in ARDS patients. We found that the difference between the two groups showed an increasing trend (an average of 0.69 per day) within 1 week after ICU admission.

The systemic inflammatory response is closely related to the occurrence and development of ARDS (2, 5). Elevated levels of inflammatory markers were associated with poor clinical outcomes of ARDS patients (21). Previous studies have shown that increased NLR including neutrophils rising and lymphocytes apoptosis, can be used as a marker of systemic inflammation to reflect the relative degree of inflammation and host immune status (22). In the latest study, the increased NLR can be used as an indicator to predict the development of ARDS in patients with COVID-19 (8). However, the physiologic mechanism behind the association between elevated NLR and poor clinical outcomes in ARDS remains unclear. We can give some possible reasons.

The literature showed that neutrophil counts are positively correlated with the degree of inflammation in ARDS (23). It has been reported that neutrophils and neutrophil-derived particles are significantly increased in bronchoalveolar lavage fluid of ARDS patients (24, 25). Several studies have shown that during the occurrence of acute lung injury (ALI), neutrophils are the first immune cells to be recruited to inflammatory sites, after being stimulated by chemokines released from damaged lung tissue. Activated neutrophils can trigger oxidative stress, release proteases, and form neutrophils extracellular traps (NETs), which induce positive feedback and further enhance the inflammatory response to lung damage (5, 23, 26). Recent studies also showed that increased neutrophils and neutrophil extracellular traps are key pathological drivers of progressive lung injury in COVID-19 patients (27). Targeting neutrophil therapy may improve the clinical outcomes of ARDS caused by SARS-COV-2 infection (19). Previous studies have shown that lymphopenia was associated with high mortality in patients with systemic inflammatory response syndrome and ARDS caused by COVID-19 (28, 29). Lymphocytes play an important role in regulating appropriate inflammatory responses, and lymphopenia may perpetuate harmful inflammation (15). Moreover, *in vitro* experiments, when lymphocytes were cultured with neutrophils, their cytolytic activity was inhibited, and the degree of inhibition increased with the addition of neutrophils (30). NLR reflects the balance between lymphocytes and neutrophils and may reflect the immune status more comprehensively. Therefore, we speculated that the dynamic elevation of NLR may reflect the severity of inflammatory response in ARDS patients, which can be an important indicator for monitoring the clinical prognosis of ARDS.

Although many studies have assessed the prognosis of ARDS patients, none of the biomarkers is perfect (31). NLR is the ratio of neutrophil count to lymphocyte count that can be obtained through routine blood tests without any additional cost, which may make it more convenient for clinical use. Early changes in NLR can be a sensitive indicator of disease severity. Therefore, we believe that dynamic monitoring of NLR after ICU admission is helpful for the early detection of patients with poor prognosis and early adoption of different treatments and nursing measures.

There were some limitations in our study. First, this was a single-center retrospective cohort study, so there was the possibility of selection bias; Second, although we have shown that the increased baseline NLR and an increasing trend over time after ICU admission were related to the poor prognosis of patients with ARDS, the causal relationship between baseline elevated NLR, early change in NLR and mortality cannot be established. So further basic research is needed. Third, the accuracy of the results may be affected due to the lack of information on the active infection (bacterial infection or viral infection) and the medication which can alter NLR levels. Fourth, as a single-center study, the results should be interpreted with caution when implicating in other populations and areas.

## Conclusion

In our study, there was a positive correlation between baseline NLR and short-term prognosis in ARDS patients. Furthermore, we found that there was a significant difference in the changes of NLR over time between non-survival and survival groups. The early increase in NLR was associated with higher short-term mortality in ARDS patients. These results suggest the potential benefit of monitoring early change in NLR for patients with ARDS. However, prospective studies are needed for further validation.

## Data Availability Statement

The original contributions presented in the study are included in the article/Supplementary Material, further inquiries can be directed to the corresponding author/s.

## Author Contributions

WZ designed the study, collected and analyzed data, and contributed to writing this manuscript. YW collected and analyzed data. GW and WL designed and supervised the study and drafted the manuscript. All authors have read and approved the final manuscript.

## Conflict of Interest

YW was employed by the company Ruibiao (Wuhan) Biotechnology Co. The remaining authors declare that the research was conducted in the absence of any commercial or financial relationships that could be construed as a potential conflict of interest.
