# Supplementary material for: The Association Between the Baseline and the Change in Neutrophil-to-Lymphocyte Ratio and Short-Term Mortality in Patients With Acute Respiratory Distress Syndrome
Source: Front Med (Lausanne). 2021 May 14;8:636869. doi: 10.3389/fmed.2021.636869 (PMC8160236; doi:10.3389/fmed.2021.636869)
Supplement: Supplementary file 1 [file Table_1.DOCX]

**The association between the baseline and the change in neutrophil-to-lymphocyte ratio and short-term mortality in patients with acute respiratory distress syndrome**

**Supplemental table 1** Subgroup analysis for the effect of baseline NLR on risk of mortality.

|  |
| --- |

|  |  | 30-day mortality | In-hospital mortality |
| --- | --- | --- | --- |
| Variables | n | OR 95% CI p-value | OR 95% CI p-value |
| Gender |  |  |  |
| Female | 475 | 1.02 (1.00, 1.04) 0.0164 | 1.02 (1.00, 1.04) 0.0113 |
| Male | 689 | 1.03 (1.02, 1.04) <0.0001 | 1.03 (1.02, 1.05) <0.0001 |
| Age |  |  |  |
| <65 | 655 | 1.03 (1.01, 1.04) 0.0011 | 1.03 (1.02, 1.05) <0.0001 |
| ≥65 | 509 | 1.02 (1.01, 1.04) 0.0047 | 1.02 (1.01, 1.04) 0.0069 |
| Smoking |  |  |  |
| No | 297 | 1.04 (1.02, 1.07) 0.0006 | 1.03 (1.01, 1.06) 0.0029 |
| Yes | 595 | 1.02 (1.00, 1.03) 0.0231 | 1.03 (1.01, 1.04) 0.0006 |
| Unknown | 272 | 1.03 (1.01, 1.05) 0.0109 | 1.03 (1.01, 1.05) 0.0117 |
| Admission type |  |  |  |
| Emergency | 1021 | 1.02 (1.01, 1.03) <0.0001 | 1.03 (1.02, 1.04) <0.0001 |
| Urgent | 94 | 1.05 (1.00, 1.11) 0.0361 | 1.05 (1.01, 1.11) 0.0303 |
| Elective | 49 | 1.05 (0.99, 1.12) 0.1192 | 1.03 (0.97, 1.09) 0.2802 |
| Ethnicity |  |  |  |
| Caucasian | 782 | 1.03 (1.01, 1.04) <0.0001 | 1.03 (1.02, 1.04) <0.0001 |
| Black | 64 | 1.01 (0.95, 1.07) 0.7687 | 1.07 (0.99, 1.16) 0.0681 |
| Hispanic | 30 | 0.85 (0.68, 1.07) 0.1616 | 0.85 (0.68, 1.07) 0.1616 |
| Others | 288 | 1.03 (1.01, 1.05) 0.0069 | 1.03 (1.01, 1.05) 0.0062 |
| Pneumonia |  |  |  |
| No | 819 | 1.02 (1.00, 1.03) 0.0131 | 1.02 (1.01, 1.03) 0.0015 |
| Yes | 345 | 1.04 (1.02, 1.07) <0.0001 | 1.05 (1.03, 1.07) <0.0001 |
| Trauma/surgery |  |  |  |
| No | 871 | 1.03 (1.02, 1.04) <0.0001 | 1.03 (1.02, 1.04) <0.0001 |
| Yes | 293 | 1.00 (0.98, 1.04) 0.7452 | 1.01 (0.98, 1.04) 0.4379 |
| Other nonpulmonary |  |  |  |
| No | 836 | 1.03 (1.01, 1.04) <0.0001 | 1.03 (1.02, 1.04) <0.0001 |
| Yes | 328 | 1.03 (1.00, 1.05) 0.0162 | 1.03 (1.01, 1.05) 0.0126 |
| Non-pulmonary sepsis |  |  |  |
| No | 615 | 1.03 (1.02, 1.05) <0.0001 | 1.03 (1.02, 1.05) <0.0001 |
| Yes | 549 | 1.02 (1.00, 1.03) 0.0190 | 1.02 (1.01, 1.04) 0.0028 |
| COPD |  |  |  |
| No | 1103 | 1.02 (1.01, 1.04) <0.0001 | 1.03 (1.02, 1.04) <0.0001 |
| Yes | 61 | 1.03 (0.99, 1.08) 0.1526 | 1.03 (0.98, 1.07) 0.2394 |
| Aspiration |  |  |  |
| No | 1087 | 1.03 (1.02, 1.04) <0.0001 | 1.03 (1.02, 1.04) <0.0001 |
| Yes | 77 | 0.99 (0.95, 1.03) 0.7168 | 0.99 (0.95, 1.03) 0.6237 |
| Diabetes mellitus |  |  |  |
| No | 892 | 1.03 (1.01, 1.04) <0.0001 | 1.03 (1.02, 1.04) <0.0001 |
| Yes | 272 | 1.02 (1.00, 1.05) 0.0419 | 1.03 (1.00, 1.05) 0.0251 |
| Hypertension |  |  |  |
| No | 1043 | 1.03 (1.01, 1.04) <0.0001 | 1.03 (1.02, 1.04) <0.0001 |
| Yes | 121 | 1.03 (1.00, 1.06) 0.0911 | 1.04 (1.01, 1.07) 0.0211 |
| Tumor |  |  |  |
| No | 1086 | 1.03 (1.02, 1.04) <0.0001 | 1.03 (1.02, 1.04) <0.0001 |
| Yes | 78 | 1.00 (0.97, 1.04) 0.8497 | 1.02 (0.98, 1.06) 0.3289 |
| Renal failure |  |  |  |
| No | 1024 | 1.02 (1.01, 1.04) <0.0001 | 1.02 (1.01, 1.04) <0.0001 |
| Yes | 140 | 1.03 (1.00, 1.06) 0.0248 | 1.05 (1.02, 1.09) 0.0007 |
| Berlin classification, n (%) |  |  |  |
| Mild | 208 | 1.02 (1.00, 1.05) 0.0804 | 1.03 (1.01, 1.05) 0.0134 |
| Moderate | 534 | 1.02 (1.01, 1.04) 0.0083 | 1.02 (1.00, 1.04) 0.0370 |
| Severe | 422 | 1.03 (1.01, 1.04) 0.0015 | 1.03 (1.02, 1.05) <0.0001 |
| Renal replacement therapy |  |  |  |
| No | 1090 | 1.03 (1.02, 1.04) <0.0001 | 1.03 (1.02, 1.04) <0.0001 |
| Yes | 74 | 1.01 (0.97, 1.04) 0.7637 | 1.03 (0.99, 1.06) 0.1262 |
| Ventilation received |  |  |  |
| No | 132 | 1.03 (0.99, 1.06) 0.1258 | 1.03 (1.00, 1.07) 0.0664 |
| Yes | 1032 | 1.03 (1.01, 1.04) <0.0001 | 1.03 (1.02, 1.04) <0.0001 |
| Antibiotic therapy |  |  |  |
| No | 615 | 1.02 (1.00, 1.03) 0.0164 | 1.02 (1.01, 1.03) 0.0074 |
| Yes | 549 | 1.04 (1.02, 1.06) <0.0001 | 1.04 (1.02, 1.06) <0.0001 |
| Corticosteroids therapy |  |  |  |
| No | 1077 | 1.02 (1.01, 1.03) <0.0001 | 1.03 (1.01, 1.04) <0.0001 |
| Yes | 87 | 1.06 (1.01, 1.10) 0.0122 | 1.07 (1.02, 1.12) 0.0029 |
| Vasopressor therapy |  |  |  |
| No | 578 | 1.05 (1.03, 1.07) <0.0001 | 1.05 (1.03, 1.07) <0.0001 |
| Yes | 586 | 1.01 (0.99, 1.02) 0.3014 | 1.01 (1.00, 1.03) 0.0620 |

P values were calculated by univariate logistic regression analysis.
